# Supplementary material for: Ultrafine Particles Issued from Gasoline-Fuels and Biofuel Surrogates Combustion: A Comparative Study of the Physicochemical and In Vitro Toxicological Effects
Source: Toxics. 2022 Dec 26;11(1):21. doi: 10.3390/toxics11010021 (PMC9861194; doi:10.3390/toxics11010021)
Supplement: Supplementary file 1 [file toxics-11-00021-s001.zip › toxics-2064784-supplementary.pdf]

Supplementary data

Ultrafine particles issued from gasoline-fuels and biofuel surrogates combustion: a comparative study of the physicochemical and *in vitro* toxicological effects

Ana Teresa Juárez-Facio[1*], Tiphaine Rogez-Florent[1+], Clémence Méausoone[1+], Clément Castilla[2], Mélanie Mignot[2], Christine Devouge-Boyer[2], Hélène Lavanant[2], Carlos Afonso[2], Christophe Morin[2], Nadine Merlet-Machour[2], Laurence Chevalier[3], François-Xavier Ouf[4], Cécile Corbière[1], Jérôme Yon[5], Jean-Marie Vaugeois[1], Christelle Monteil[1].

[1] UNIROUEN, Université de Rouen Normandie, ABTE, 76000 Rouen, France

[2] UNIROUEN, INSA Rouen, CNRS, COBRA, 76000 Rouen, France

[3] UNIROUEN, INSA Rouen, CNRS, GPM-UMR6634, 76000 Rouen, France

[4] Institut de Radioprotection et de Sureté Nucléaire, PSN-RES, SCA, LPMA, Gif-sur-Yvette, France

[5] UNIROUEN, INSA Rouen, CNRS, CORIA, 76000 Rouen, France

***Corresponding author email:** anatere.jf@gmail.com (Ana Teresa Juarez-Facio)

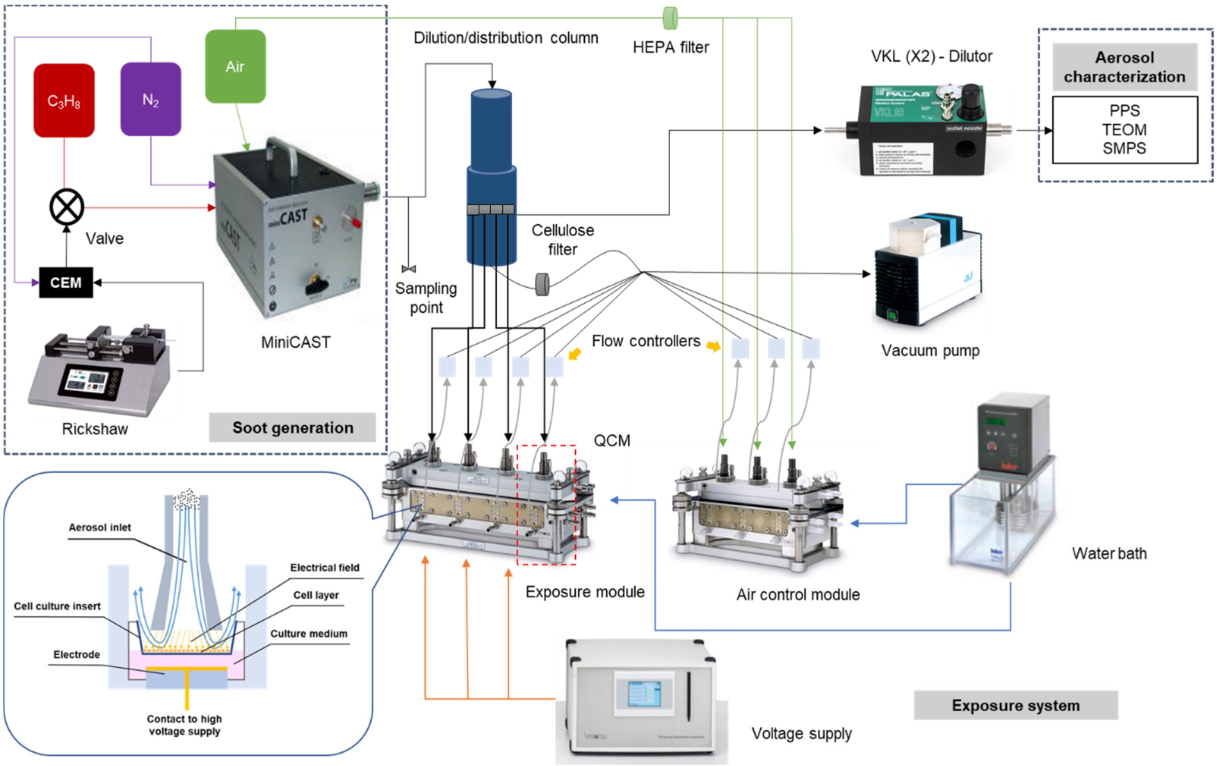

Figure S1: Aerosol exposure system. Adapted from (Juarez-Facio et al., 2022)

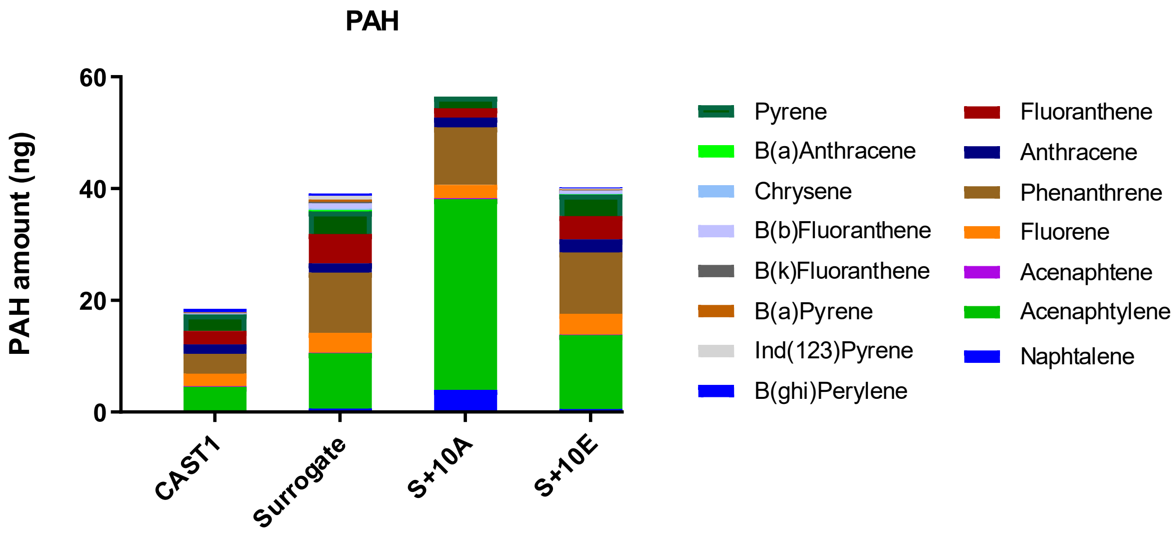

Figure S2: Amount of the different PAH and oxy-PAH in the four studied aerosols.

Table S1: LDI+ FTICR MS method parameters

MALDI			Acquisition		
	Plate Offset (V)	100		Low m/z	98.27
	Deflector Plate (V)	200		High m/z	1200
	Laser power (%)	18		Size	4M
	Laser Shots	100		Average scans	30
	Frequency (Hz)	1000		Accumulation (s)	x
	Laser Focus	Medium		FID (s)	1.25835
**Ion Transfer**			**Analyzer**		
Source optics	Capillary Exit (V)	150	Para Cell	Transfer Exit Lens (V)	-20
	Deflector Plate (V)	200		Analysis Entrance (V)	-10
	Funnel 1 (V)	150		Side Kick (V)	0
	Skimmer 1 (V)	25		Side Kick Offset (V)	-1.5
	Funnel RF Amplitude (Vpp)	60		Front Trap Plate (V)	1.7
Octopole	Frequency (MHz)	5		Back Trap Plate (V)	1.7
	RF Amplitude (Vpp)	350		Back Trap Plate Quench (V)	-30
Quadrupole	Q1 Mass(m/z)	120		Sweep Excitation Power (%)	35
Collision Cell	Collision Voltage (V)	0.5	Shimming DC Bias	0°	1.513
	DC Extract Bias (V)	0.5		90°	1.51
	RF Frequency (MHz)	2		180°	1.487
	Collision RF Amplitude (Vpp)	1200		270°	1.49
Transfer Optics	Time of Flight (ms)	0.8	Gated Injection DC Bias	0°	2.3
	Frequency (MHz)	6		90°	1.5
	RF Amplitude (Vpp)	200		180°	0.7
				270°	1.5

Table S2: Internal recalibration ions for LDI+ FTICR MS analysis

Ion	m/z	Ion	m/z
**C19H11**	239.08553	C42H18	522.14030
**C20H12**	252.09335	C45H19	559.14813
**C21H13**	265.10118	C46H20	572.15595
**C22H12**	276.09335	C48H20	596.15595
**C23H13**	289.10118	C50H20	620.15595
**C25H13**	313.10118	C52H20	644.15595
**C26H14**	326.10900	C53H21	657.16378
**C28H14**	350.10900	C55H21	681.16378
**C29H15**	363.11683	C58H22	718.17160
**C31H15**	387.11683	C60H24	744.18725
**C33H15**	411.11683	C62H24	768.18725
**C34H16**	424.12465		
**C37H17**	461.13248		
**C40H18**	498.14030		

Table S3: Estimated concentrations for the compounds detected on the gas phase, sampled with and without filter (/: not detected).

		**CAST1**		**Surrogate**		**S+10A**		**S+10E**		**Limit of quantification**
	Compounds	*Molar % (with filter)*	*Molar % (without filter)*	*Molar % (with filter)*	*Molar % (with filter)*	*Molar % (with filter)*	*Molar % (without filter)*	*Molar % (with filter)*	*Molar % (without filter)*	*Molar %*
TCD	Dihydrogen H_2	/	/	0,034	0,03	0,03	0,031	0,035	0,033	0.008
	Methane CH_4	/	/	0,027	0,029	0,029	0,03	0,035	0,036	0.035
MS	Carbon dioxide CO_2	1,0085	0,8539	0,6305	0,6559	0,6559	0,6059	0,5827	0,6379	0.0044
	Ethylene C_2H_4	/	/	0,0159	0,0183	0,0183	0,0164	0,0227	0,0254	0.0005
	Acetylene C_2H_2	/	/	0,0104	0,0119	0,0119	0,0105	0,0135	0,0160	0.0119
	Ethane C_2H_6	/	/	0,0007	0,0008	0,0008	0,0005	0,0008	0,0010	0.0196
	Propene C_3H_6	/	/	0,0045	0,0035	0,0035	0,0031	0,0039	0,0041	0.0003
	Propane C_3H_8	0,0005	0,0002	/	/	/	/	/	/	0.0003
	Propadiene C_3H_4	/	/	0,0012	0,001	0,001	0,0009	0,0011	0,0011	0.0003
	Propyne C_3H_4	/	/	0,0022	0,002	0,002	0,0017	0,0021	0,0023	0.0003
	Acetaldehyde C_2H_4O	/	/	/	/	/	/	0,0001	0,0001	0.0003
	Isobutene C_4H_8	/	/	0,0013	0,001	0,001	0,0008	0,0011	0,0011	0.0003
	1-buten-3-yne C_4H_4	/	/	0,0007	0,0009	0,0009	0,0006	0,0009	0,0010	0.0003
	1,3 butadiene C_4H_6	/	/	0,0022	0,0022	0,0022	0,0019	0,0025	0,0027	0.0003
	1,3 butadiyne C_4H_2	/	/	/	/	/	/	0,0002	0,0002	0.0003
	Cyclopentadiene C_5H_6	/	/	0,0011	0,0023	0,0023	0,0016	0,0017	0,0022	0.0003
	Benzene C_6H_6	/	/	0,00004	0,00004	0,00004	0,00004	0,00004	0,00004	0.00002
	Toluene C_7H_8	/	/	0,00004	0,00004	0,00004	0,00004	0,00004	0,00006	0.00002
